# Warpage Reduction of Glass Fiber Reinforced Plastic Using Microcellular Foaming Process Applied Injection Molding

**DOI:** 10.3390/polym11020360

**Published:** 2019-02-19

**Authors:** Hyun Keun Kim, Joo Seong Sohn, Youngjae Ryu, Shin Won Kim, Sung Woon Cha

**Affiliations:** School of Mechanical Engineering, Yonsei University, 50, Yonsei-ro, Seodaemun-gu, Seoul 03722, Korea; sagegny@yonsei.ac.kr (H.K.K.); ssamjjang87@yonsei.ac.kr (J.S.S.); yjryu1027@yonsei.ac.kr (Y.R.); 0shinmy0@yonsei.ac.kr (S.W.K.)

**Keywords:** fiber orientation, cell morphology, injection molding, microcellular foaming process, warpage, shrinkage, mechanical properties

## Abstract

This study analyzes the fundamental principles and characteristics of the microcellular foaming process (MCP) to minimize warpage in glass fiber reinforced polymer (GFRP), which is typically worse than that of a solid polymer. In order to confirm the tendency for warpage and the improvement of this phenomenon according to the glass fiber content (GFC), two factors associated with the reduction of the shrinkage difference and the non-directionalized fiber orientation were set as variables. The shrinkage was measured in the flow direction and transverse direction, and it was confirmed that the shrinkage difference between these two directions is the cause of warpage of GFRP specimens. In addition, by applying the MCP to injection molding, it was confirmed that warpage was improved by reducing the shrinkage difference. To further confirm these results, the effects of cell formation on shrinkage and fiber orientation were investigated using scanning electron microscopy, micro-CT observation, and cell morphology analysis. The micro-CT observations revealed that the fiber orientation was non-directional for the MCP. Moreover, it was determined that the mechanical and thermal properties were improved, based on measurements of the impact strength, tensile strength, flexural strength, and deflection temperature for the MCP.

## 1. Introduction

Plastic is cheap and has outstanding material properties for its density. Furthermore, it is easy to manufacture, so it is easy to use for the production of various components, which is why it is widely used in industry. Moreover, research on plastics is ongoing and their material properties have been enhanced to allow their use in areas that plastics could not be utilized otherwise because of their poor properties.

One current research direction is focused on enhancing the material properties of plastics to make them suitable for new applications, which has resulted in the development of fiber reinforced plastic. Fiber reinforced plastic is produced by reinforcing a plastic with glass fibers, carbon fibers, natural fibers, or similar material inside of the plastic. Injection products made of fiber reinforced polymers have especially higher mechanical strength and heat-resistance compared to the existing resins. Moreover, as injection molding can be applied to other materials similar to the existing resins without additional processes, fiber reinforced plastics have high applicability and are used widely in the industry. Injection molding is especially popular in industries such as automobile parts and electronics manufacturing, where parts with higher material properties are increasingly required. Fiber reinforced plastic injection products are being used in more areas [1,2].

The problem with fiber reinforced plastic products is the warpage that is generated after product blowdown from the mold during the injection molding process. In particular, the fiber reinforced plastic’s fiber orientation intensifies this warpage [3,4,5,6,7]. In this work, to overcome the warpage of fiber reinforced plastic, the microcellular foaming process (MCP) was used.

The microcellular foaming process is a plastic foaming process, in which micropores are formed inside the plastic. The MCP results in smaller cell sizes than the existing foaming processes, as well as a high cell density. Through the use of the MCP, the plastic is lightened by 10%~50 %, and its material properties are enhanced [8,9,10,11,12,13]. In addition, by applying the MCP, process cycle time can be decreased by decreasing the hold and packing time, and with the decrease in viscosity, the process temperature could be decreased. As a result, the application of the MCP decreases the amount of plastics used and it could be said to be an environment-friendly process because the amount of energy used in the process can be decreased [14,15]. In this study, the MCP was applied to address the issue of warpage of GFRP. Polyproplyene was selected as the base material and glass fiber reinforced specimens were prepared. We confirmed that the warpage was exacerbated when glass fiber was added and experiments were conducted in an attempt to improve the warpage of the specimen by utilizing the MCP. In a previous investigation on warpage, Baek and Lee [16] studied the warpage characteristics of film insert molded parts according to the injection molding process conditions. Their research investigated warpage characteristics according to process conditions such as temperature, speed, and pressure, and it corresponds to insert injection process without the MCP. Kramschuster and Cavitt [17] studied the shrinkage and warpage characteristics of the injection process with the MCP. They confirmed the reduction of shrinkage and warpage in the MCP via a quantitative investigation. Although the supercritical fluid (SCF) content and the injection speed were determined to have the greatest influence on warpage and shrinkage, neither the relationship between shrinkage and warpage nor the cause of the decrease was addressed. Jonathan and Park also studied GFRP using the MCP [18]. They investigated warpage and shrinkage according to process conditions such as injection speed, gas contents, and shot size. In addition, they examined shrinkage characteristics according to cell density. However, they were only able to confirm that shrinkage improves as the cell density increases. Sadabadi and Ghasemi [19] studied fiber orientation according to the process conditions in short glass fiber polystyrenes and investigated the change of fiber orientation according to the process conditions. In order to verify the compatibility of GFRP with the MCP, physical and thermal properties were measured. Bledzki and Kirschlig [20] examined the physical properties of polycarbonate when the MCP was utilized, based on the process conditions. Lin and Zhang [21] studied the physical properties of foams with short fibers and rubbers. As previously indicated, existing studies are focused on the minimization of warpage via the control of process conditions. However, there is a lack of research on the analysis of the underlying principle or cause of this phenomenon. In this study, we focused on identifying and verifying the cause of warpage reduction when the MCP is utilized as opposed to the reduction of warpage based on changes in the process conditions. Moreover, the correlation between warpage and shrinkage is not considered as a separate phenomenon. As a result, it was confirmed that the shrinkage difference according to direction is closely correlated with warpage. It was confirmed that the MCP minimizes warpage as a result of a reduction of the shrinkage difference. We also identified two causes of shrinkage difference reduction that are related to the decrease in the absolute value of the shrinkage due to cell growth and the randomization of the fiber orientation.

## 2. Experimental

### 2.1. Material

In this experiment, the SUPRAN resin from Lotte Chemical (Seoul, Korea) was used. The SUPRAN resin is a compound of polypropylene and glass fibers. The polypropylene base resin that was used in the SUPRAN resin is LOTTE Chemical’s J-150 grade and its density was measured according to ASTM D 792 and its value was found to be 0.90 g/cm^3^. Its melting index was measured according to ASTM D 1238 and the resin with a melting index of 10 g/10 min was used. Resins with glass fiber content in the range of 0%~60% in 10% increments were used to determine how the material properties change according to the glass fiber content. Nitrogen (N_2_) was used as a foaming gas and this gas remained in the SCF state.

The polypropylene glass fiber mixture has a relatively low process temperature and has high moldability. In addition, it has outstanding mechanical strength for its density and its price is cheap which makes it one of the most widely used fiber reinforced plastics in the industry. However, polypropylene has a low glass transition temperature of −10 °C. Because the warpage characteristics, which are caused by the fiber reinforced plastic’s low glass transition temperature, can clearly be observed, this material was selected for this research.

### 2.2. Microcelluar Foaming Process Applied to Injection Molding

**Figure 1:**
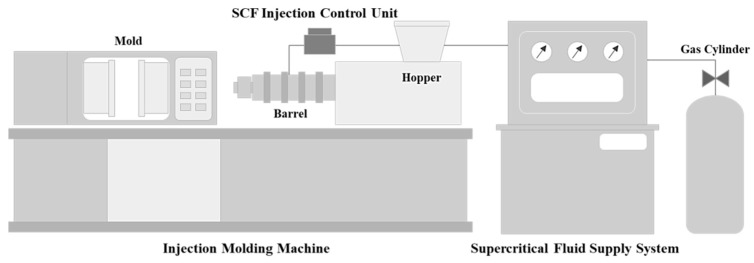
Microcellular foaming process applied to the injection molding process.

The process used in this research is the injection process that uses the microcellular foaming process. Figure 1 shows a schematic diagram of the injection molding equipment that was used in this research. This equipment applies the MCP to the injection molding process. It injects the gas inside of the polymer fluid that is kept in a liquid state by the temperature inside of the barrel and the friction heat from the gas in the supercritical fluid (SCF) state that was injected by the SCF supply system. Inside of the barrel in the high-temperature and high-pressure state, the melted polymer and gas are mixed into a solution. Due to the rate of pressure decrease during injection, thermodynamic instability is created to induce cell nucleation, and in the process of filling the mold, a porous structure is molded as the cells grow.

### 2.3. Warpage and Shrinkage

**Figure 2:**
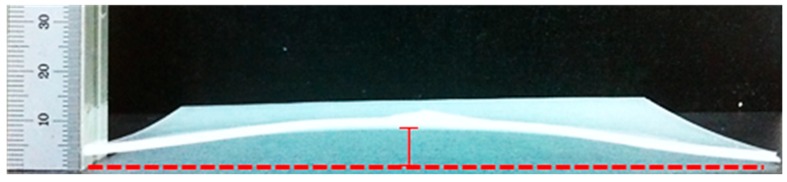
Method of measuring the warpage of a specimen using height meter.

Because warpage does not have an ASTM size, the warpage was measured using the warpage measurement methods used in previously published research. Using a specimen on a plate, the maximum height of the plate specimen when warpage occurs was measured using a digital height meter according to the ASTM D 5947 standard of the solid plate in order to measure the warpage (Figure 2). For the warpage specimen, specimens with even warpage formation were used to measure the absolute value of the warpage [22].

The shrinkage value was determined based on the ratio of how much the specimen contracted in comparison to the actual mold according to the ASTM D 955 standard. The size of the specimen used was 128 mm × 12.48 mm × 3 mm and the flow shrinkage, which is the shrinkage in the flow direction, and the transverse shrinkage, which is the shrinkage perpendicular to the flow direction, were each measured. For the shrinkage specimen, a specimen with a propensity for fibers to be oriented in one direction with its fiber orientation tensor value as close to 1 as possible and a specimen that shows clear contraction deviations according to the fiber orientation were used to identify the contraction deviations from the shrinkage and the flow direction of the resin.

### 2.4. Cell Morphology

In this investigation, a morphology analysis was conducted for a cell. Based on this analysis, the effect of cell growth on shrinkage reduction was confirmed. Moreover, the effects of cell formation on the orientation of the fiber were also identified during the MCP. Scanning electron microscope (SEM) images were used to observe cell morphology. The cell morphology was quantified by measuring the formed cells’ sizes and the cell density was determined by calculating the number of cells per unit volume.

### 2.5. Fiber Orientation 

In order to identify the changes in fiber orientation, the fiber orientation’s angle distribution was measured using Micro-CT imaging. For the fiber orientation, Bruker AXS’s Micro CT (Bruker Co., Billerical, Model No. Skyscan 1172, MA, USA) was used to take the images. CT-analyzer software (Bruker) was used for CT image analysis. The imaging was conducted with a pixel size of 13.4 μm. With the measured orientation of the fiber, the image analysis tool, ImageJ, was used to measure the fiber’s angle with respect to the x-axis to compute an angular distribution. The measured specimen was imaged using a part of the plate specimen that was used to measure the warpage, and by imaging the solid specimen and the MCP-applied specimen at the same location, the fiber orientations of each were compared [23,24].

### 2.6. Mechanical Properties 

The mechanical properties of the specimens were measured, including impact strength, flexural strength, and tensile strength. The impact strength test was performed on an IZOD notched specimen using the IZOD impact strength tester (Salt Co., Ltd., Model No. ST-120, Incheon, Korea). The flexural strength test and tensile strength test were conducted with a universal testing machine (QMESYS Co., Ltd., Model No. QM-100T, Gunpo, Korea). The impact strength was measured in accordance with the ASTM D 256 method using specimens with dimensions of 63.5 × 12.7 × 3.0 (mm^3^). The impact was applied to the side of the notch [25] and impact strengths were calculated after five repeated measurements. The tensile strength was measured in accordance with the ASTM D 638 method for specimens with dimensions of 165 × 19 × 3.3 (mm^3^). The cross-head speed was 10 mm/min during the measurement of the tensile strength [26]. Tensile strength values were determined after five repeated measurements. In the case of the flexural strength, values were measured in accordance with the ASTM D 790 method and the specimen sizes were 127 × 12.7 × 6.4 (mm^3^). The support distance of the specimens was 102.4 mm and the test load speed was 10 mm/min [27]. Flexural strength were calculated after five repeated measurements.

### 2.7. Deflection Temperature 

To identify the impact of MCP application to the heat-resistance, which is one of the advantages of fiber reinforced plastics in comparison to regular plastics, the deflection temperature was measured. The deflection temperature is measured by increasing the temperature at a fixed load until deflection occurs. In this work, the ASTM D 648-07 Standard Test Method for Deflection Temperature of Plastics Under Flexural Load in the Edgewise Position was used to measure the deflection temperature. Specimens with dimensions of 127 mm × 12.5 mm × 3 mm were used to measure this value. Maintaining a fixed load of 1.82 MPa and increasing the temperature by 2 ± 0.2 °C/min, the temperature was measured until the deflection reached 0.25 mm.

## 3. Results and Discussion

### 3.1. Warpage Reduction of MCP-Applied Glass Fiber Reinforced Plastic

The tendency of the warpage to decrease was identified when the MCP was applied to the fiber reinforced plastic. The experiments were conducted with uniform injection pressure, injection speed, and injection temperature during injection molding, and the foaming ratio (Equation (1)) was controlled by adjusting the amount of SCF used during the injection molding. The foaming ratio, as shown in Table 1, was calculated by measuring the solid specimen’s density and the foaming specimen’s density. The experiment was conducted with foaming ratios of 5 %, 10 %, and 15 % to measure the decrease in warpage as a function of the foaming ratio.

(1) $$\mathrm{Foaming}\;\mathrm{Ratio}\left(\%\right)=\frac{d_{0}-d_{1}}{d_{0}}\left(\%\right)$$

$d_{0}$ = Density of Solid Specimen; $d_{1}$ = Density of Foamed Specimen.

**Figure 3:**
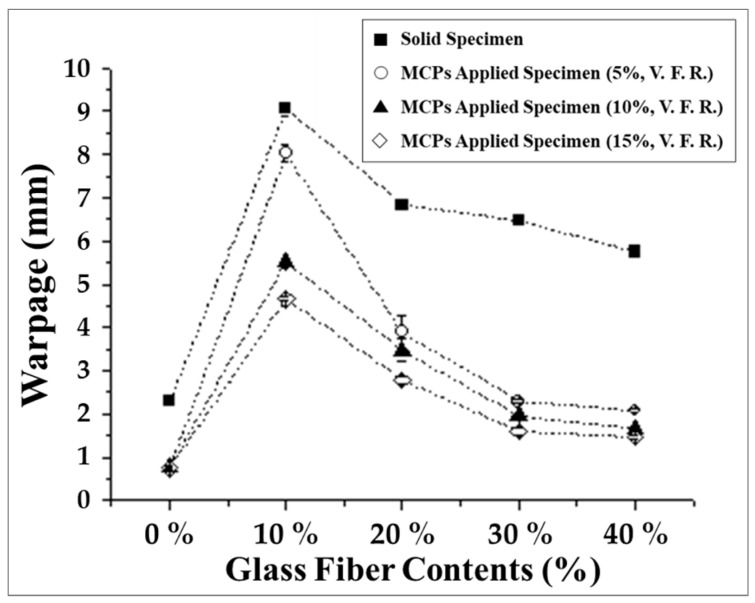
Warpage of microcellular foaming process (MCP)-applied glass fiber reinforced plastics.

As a result, the changes in warpage when the MCP is applied are shown in Figure 3. As the foaming ratio was increased, the warpage decreased. Through these experiments, the warpage reducing effects of the MCP were confirmed and the relationship between the warpage and glass fiber content and foaming ratio were identified. Furthermore, it was determined that the warpage could be reduced by 70 % just by applying the MCP without altering any other process conditions.

### 3.2. Shrinkage

**Figure 4:**
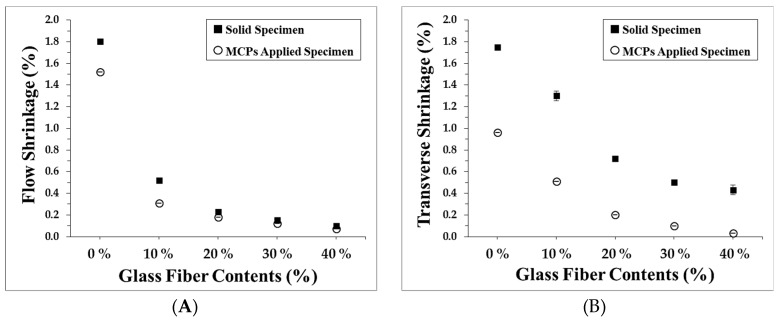
Shrinkage of specimens: (**A**) Flow shrinkage and (**B**) Transverse shrinkage.

To quantify the shrinkage, the flow shrinkage, which is the contraction of the resin in the flow direction, and the transverse shrinkage, which is the shrinkage perpendicular to the flow direction, were each measured. During these experiments, the foaming ratio was kept constant at 15%. The glass fiber contents were varied in the range 0%–40%. The flow shrinkage as a function of glass fiber content is shown in Figure 4A and the transverse shrinkage is shown in Figure 4B. From an examination of the flow shrinkage and the transverse shrinkage behavior of the MCP-applied specimens, the reasons for the warpage reduction when the MCP is applied could be identified indirectly. For the flow shrinkage, although the flow shrinkage value decreases when the MCP is applied, the changes are relatively small. On the other hand, the transverse shrinkage showed relatively large reductions. The warpage varies according to the deviations in fiber orientation. The flow shrinkage is reduced while the transverse shrinkage is reduced relatively less, which leads to warpage from the contraction deviations. When the MCP is applied, as the flow shrinkage, which naturally has a relatively small value, is reduced a little and the transverse shrinkage, which typically has a high value, shows a higher reduction rate, it works to effectively reduce the deviation.

### 3.3. Cell Morphology

**Figure 5:**
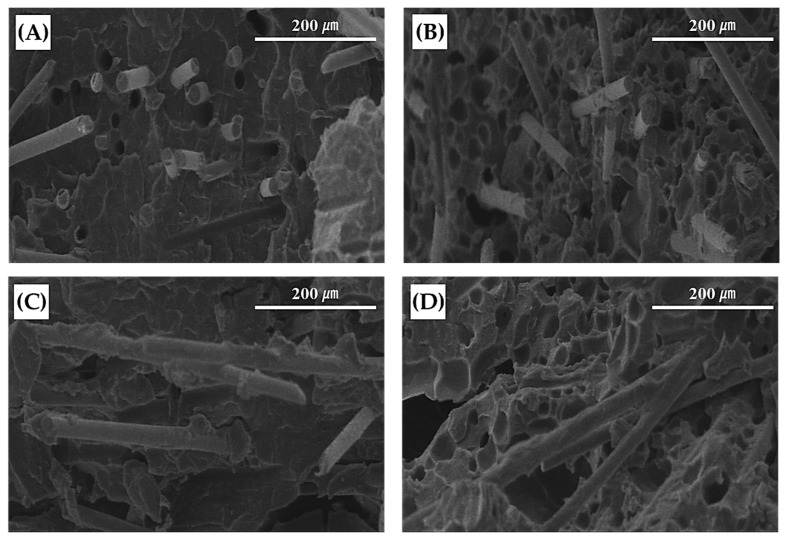
Scanning electron microscopy images for cell morphology analysis (magnification: 200×): (**A**) front view of solid specimen, (**B**) front view of MCP-applied specimen, (**C**) side view of solid specimen, and (**D**) side view of MCP-applied specimen.

The morphology of the cells formed by the MCP was examined using a scanning electron microscope (JEOL Ltd., Product No. 7001F, Tokyo, Japan) (Figure 5). After applying the MCP to the fiber reinforced plastic with a glass fiber content of 20%, because over 20% of the samples exhibited significant warpage effect. Moreover, a sample ratio of fiber to cell of 20% was sufficient to facilitate observation of the cell’s morphology (Table 2). The analysis of the cell morphology of the specimen with a void fraction ratio of 15 % showed an average cell size of 15.4 μm and a cell density of 3.1 × 10^7^ cells/ cm^3^. From the cells formed in such ways, the absolute reduction in the shrinkage could be identified. The gas that was dissolved inside of the polymer in the high-temperature, high-pressure barrel is eluted and forms cells, while inside of the formed cells, energetic inert gases are trapped in the cells, giving them volume and pressure. At this moment in the cell growth process, this internal pressure applies force to the cell surface. This outward force cancels the thermal shrinkage that would cause the cells to contract, thus decreasing the absolute value of the shrinkage. Through the SEM images, the even and uniform formation of cells could be identified and the fiber orientation’s changes could be identified indirectly.

### 3.4. Fiber Orientation

**Figure 6:**
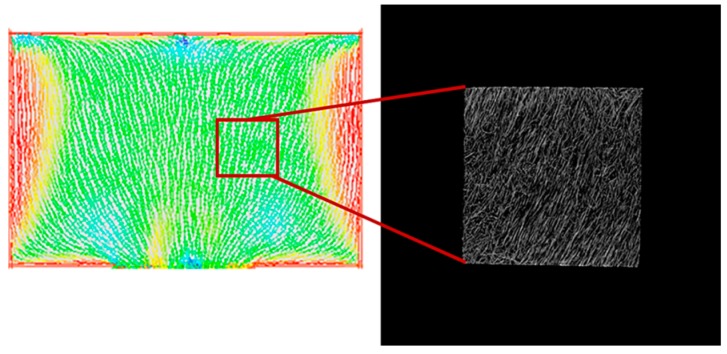
Comparison of moldflow CAE with Micro-CT image.

**Figure 7:**
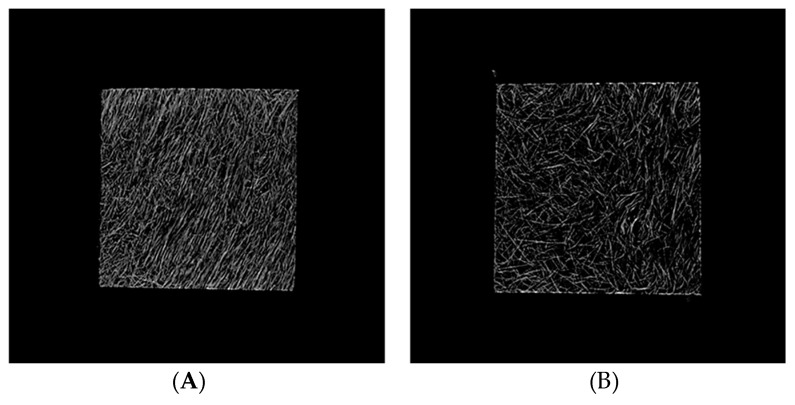
Micro-CT images of plate specimens: (**A**) solid specimen and (**B**) MCP-applied specimen.

**Figure 8:**
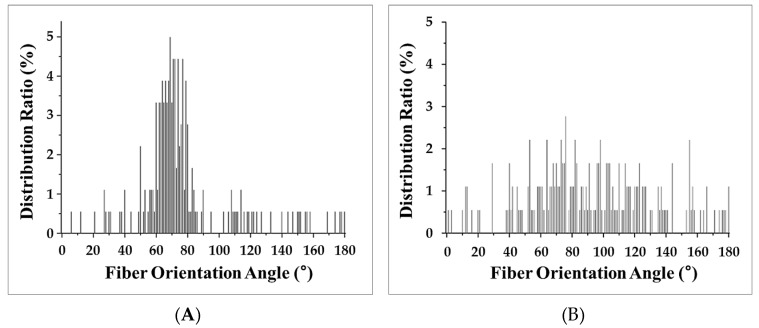
Fiber orientation angle distribution: (**A**) solid specimen and (**B**) MCP-applied specimen.

To image the changes in fiber orientation, Micro CT imaging was conducted. A solid specimen formed without the MCP and MCP-applied specimens were imaged with the Micro CT tool to conduct this experiment. The analysis of the fiber orientation used a part of the plate specimen that was used to measure the warpage for imaging. Parts of the solid specimen and the plate specimen were used for imaging. As shown in Figure 6, the Micro CT imaging shows that the result of the Mold Flow (CAE Software, Autodesk Inc., San Rafael, CA, USA) analysis and the actual image of the fibers are quite similar. Figure 7 shows the results of the analysis of the fiber orientations of the solid specimen and MCP-applied specimen in (A) and (B), respectively. As the result of the image analysis, the solid specimen’s fiber orientation with a fixed direction and the relatively randomized fiber orientation of the MCP-applied specimen could be identified with the naked eye. In order to quantify the fiber orientation, the fiber orientation distributions were calculated using the image analysis program, ImageJ. The results of the fiber orientation distribution analysis for the solid specimen and the MCP-applied specimen are shown in Figure 8A,B, respectively. For the solid specimen, the fibers were concentrated at an orientation of 70°, as shown in the Micro CT image, while the fibers of the MCP-applied specimen were more widely distributed compared to the solid specimen. To compare the standard deviation value of the fiber orientation angle, the solid specimen exhibited a value of 29.68, and the MCP-applied fiber orientation angle had a standard deviation value of 39.48, showing an increase of over 24%. Due to this, when the MCP was applied, the fibers that used to be concentrated were randomized, which resulted in a reduction of warpage due to the fiber orientation.

### 3.5. Mechanical Properties

**Figure 9:**
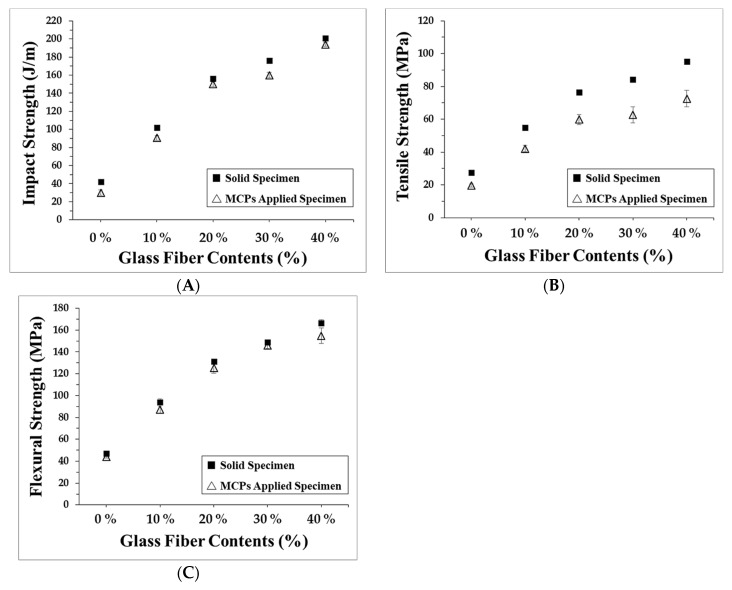
Mechanical properties: (**A**) impact strength, (**B**) tensile strength, and (**C**) flexural strength.

The warpage reduction when the MCP is applied was measured and the warpage characteristics reduced by the used of the MCP were identified and research was conducted to understand the reasons for this reduction. Furthermore, the material properties of the MCP-applied specimen were measured in order to confirm whether the MCP-applied specimen could actually be used in industrial applications. These properties were measured through the impact strength test, tensile strength test, flexural strength test, and the heat deflection temperature test. Specimens with glass fiber contents of 0%~40 % and a foaming ratio of 15% were used for these measurements. The results are shown in Figure 9. The measured properties of the MCP-applied specimens were found to be decreased in comparison to the solid specimen. This allows us to identify the decrease in the material properties with the stress concentration from the gas bubbles that are formed inside of the MCP-applied specimens. However, considering the fact that the plastic is lightened by 15% when the MCP is applied, these plastics could be applied to actual products despite their decreased material properties, especially as the interest in lighter products is increasing. Moreover, in the case of the strength that the material withstands per share, since the MCP-applied specimens exhibited higher strength than the solid specimens, advantages of the MCP for product applications could be identified. In addition, as the heat deflection temperature was reduced by only a small amount, the MCP-applied specimens could maintain good heat-resistance, which is one of the advantages of fiber reinforced plastics.

### 3.6. Deflection Temperature

**Figure 10:**
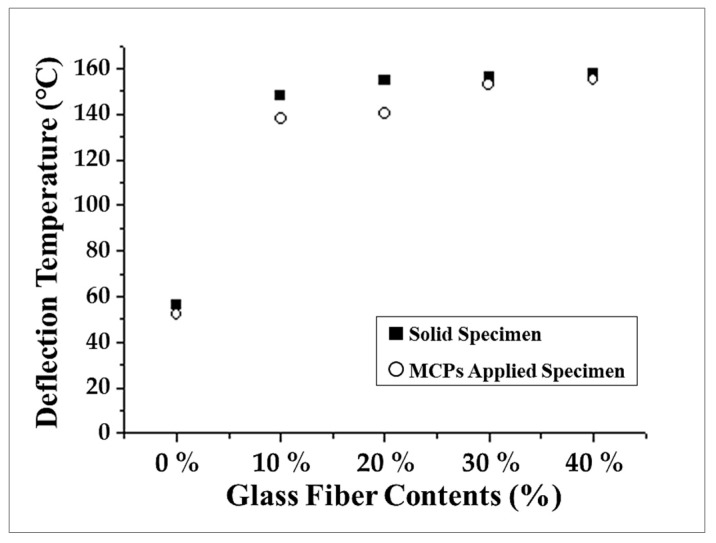
Deflection temperature of specimens.

Compared to normal plastics, one of the advantages of fiber reinforced plastic is its heat-resistance. Compared to a normal plastic, which has restricted application areas because of its low heat-resistance, fiber reinforced plastics are expanding their application areas because of their higher heat-resistance. For parts that are sensitive to temperature, such as vehicle parts and electronic parts, heat-resistance is important. In order to identify the changes in heat-resistance when the MCP is applied, the heat deflection temperature was measured. To measure the heat deflection temperature as a function of the glass fiber content, the deflection temperatures of solid specimens and MCP-applied specimens were measured. The results are shown in Figure 10. For deflection temperature, fiber reinforced plastics showed significantly higher values than the normal plastics with a glass fiber content of 0%. When the MCP was applied, the deflection temperature tended to decrease slightly, but taking into consideration the 15% lightening and the warpage reduction, this slight reduction in deflection temperature can be tolerated.

## 4. Conclusions

Based on this study, we confirmed the warpage reduction effect of GFRP when the MCP was utilized. When the warpage tendency was examined according to the glass fiber content (0~40 %), it was confirmed that the addition of glass fiber causes a warpage of the composite. In particular, when the glass fiber content was 10%, the largest warpage corresponding to a value of about 9.1 mm was observed. The cause of the larger warpage in GFRP was considered to be attributed to the difference in shrinkage and experiments were conducted to confirm this assertion. The flow and transverse shrinkage were measured and the difference between the two values was analyzed. Based on this analysis, it was confirmed that shrinkage difference and warpage have a similar tendency. In addition, a warpage reduction effect was confirmed by applying the MCP to each specimen. In order to confirm the cause of the minimization of the warpage when the shrinkage difference of the MCP was reduced, the observed tendency was confirmed and the cause was identified by measuring the shrinkage in the flow and transverse directions of the specimens before and after the MCP. It was considered that there are two main reasons why the MCP improves warpage via reduction of shrinkage differences. Therefore, we performed analysis of cell morphology and fiber orientation. It was confirmed that the shrinkage difference according to direction is closely correlated with warpage. It was confirmed that the MCP minimizes warpage as a result of a reduction of the shrinkage difference. We also identified two causes of shrinkage difference reduction that are related to the decrease in the absolute value of the shrinkage due to cell growth and the randomization of the fiber orientation. As a result, this study confirmed that warpage, which is a critical issue for GFRPs, can be significantly improved via the MCP.

In order to verify the compatibility of GFRPs with the MCP, mechanical properties including impact, tensile, and flexural strength were measured. It was therefore confirmed that the specimens that used the MCP exhibited improved properties compared to the unfoamed solid samples. In addition, by measuring the deflection temperature, it was also confirmed that GFRPs that utilized the MCP had excellent thermal properties. This is one of the merits when glass fiber is added. Furthermore, if other advantages of the MCP, such as reduced weight, improved noise shielding properties, improved optical reflection properties [28,29], and reduced process energy are considered in addition to the improvement in warpage, it will be even more promising.

The results of this study can be summarized as follows: it is confirmed that the warpage phenomenon which is exacerbated in GFRP can be improved using the MCP; it was also confirmed that warpage reduction has a similar tendency to shrinkage difference according to direction and it was also established that the MCP improves warpage by reducing the shrinkage difference via reduction of the absolute shrinkage value and fiber orientation distribution deviation by cell growth; finally, it was confirmed that the thermal properties of GFRPs have better compatibility with existing solid polymer when the MCP is utilized.

## Figures and Tables

**Table 1:** Results based on foaming ratio equation.

Variable		Value			
**Glass Fiber Content (%)**		0%	10%	20%	30%
**d_0_ (g/cm^3^)**	**Solid**	0.91	0.97	1.02	1.09
**d_1_ (g/cm^3^)**	**A**	0.86	0.92	0.97	1.04
	**B**	0.82	0.87	0.92	0.98
	**C**	0.77	0.82	0.87	0.93
**Foaming Ratio (%)**		A: 5%, B: 10%, C: 15%			

**Table 2:** Summary of effects of cell morphology on shrinkage and fiber orientation.

Classification	Effects	
**Test Condition**	Glass Fiber Contents (%)	20
	Void Fraction Ratio (%)	15
**Cell Morphology**	Cell Size (μm)	15.4
	Cell Density (cells/cm^3^)	3.1 × 10^7^
**Effects of Cell**	Shrinkage	Cell growth: Reduced
	Fiber Orientation	Oriented Non Oriented
